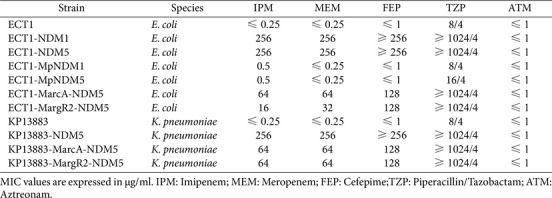# Highly Conserved Genetic Factors Regulating *bla*_NDM_ Gene Expression

**DOI:** 10.4014/jmb.2412.12081

**Published:** 2025-07-14

**Authors:** Jianfen Xu, Jinnuo Zhu, Changqing Mei, Xiaopeng Liu, Zhiming Gong, Jiansheng Huang, Hui Chai

**Affiliations:** 1 School of Life Sciences, Zhejiang Chinese Medical University, Hangzhou, Zhejiang, P.R. China; 2 Department of Clinical Laboratory, The Fifth Affiliated Hospital of Wenzhou Medical University, Lishui, Zhejiang, P.R. China

**Keywords:** *bla*_NDM_ gene, carbapenem resistance, conserved sequence, gene regulation

## Abstract

The New Delhi metallo-β-lactamase (NDM; EC 3.5.2.6) poses significant challenges to carbapenem treatment, yet the regulatory mechanisms governing *bla*_NDM_ gene expression remain poorly understood. In this study, we identified a highly conserved 110 bp sequence located upstream of the *bla*_NDM_ gene by comparative analysis of 109 clinical isolates and 2,706 nucleotide sequences from the NCBI database. This conserved sequence was characterized in all available NDM variants spanning 80 bacterial species. Bioinformatics analysis revealed a core promoter (PNDM) and two transcription factor binding sites (ArcA and ArgR2) within the sequence. Mutations of the PNDM promoter significantly reduced *bla*_NDM_ expression (mRNA and protein) by more than 90% (*P* < 0.01) and completely restored carbapenem susceptibility. Electrophoretic mobility shift assays (EMSA) confirmed the specific binding of ArcA and ArgR2 proteins to their predicted sites. Mutations in the ArcA and ArgR2 binding sites decreased *bla*_NDM_ protein production with less pronounced effects compared to PNDM promoter mutations, resulting in 24% and 32.7% reductions, respectively. The findings highlight the critical role of the highly conserved 110 bp sequence in regulating *bla*_NDM_ expression, offering potential targets for novel therapeutic strategies.

## Introduction

NDM is a clinically significant metallo-β-lactamase (MBL) capable of hydrolyzing a broad spectrum of β-lactam antibiotics, including carbapenems [1]. In China, NDM has become the second most prevalent carbapenemase gene, surpassed only by *Klebsiella pneumoniae* carbapenemase (KPC), thus significantly narrowing the range of effective antibiotics [2]. Since the initial identification of NDM-1 in *K. pneumoniae* in 2008, over 70 variants have been documented (available at: https://www.ncbi.nlm.nih.gov/pathogens/refgene/#*bla*_NDM_), with NDM-1 and NDM-5 being the most prevalent and widely disseminated [3]. NDM-1 and NDM-5 exhibit adaptive evolution under selective pressures, such as zinc deficiency, facilitating the persistence of resistance genes [4]. Although peptide analogs such as PEP4 can enhance the efficacy of imipenem by binding to the catalytic pocket of NDM, mutations such as M154L in NDM-5 enhance carbapenem hydrolysis, rendering inhibitors like PEP4 ineffective [5, 6]. Therefore, developing a regulatory strategy applicable to various NDM variants could facilitate the creation of broad-spectrum inhibitors.

While studies on *bla*_KPC_ genes highlight the significance of multiple promoters and non-coding regions [7], the regulatory mechanisms governing the upstream regions of *bla*_NDM_ genes remain largely unexplored. One well-characterized feature of the *bla*_NDM_ upstream regions is their frequent association with the insertion sequence ISAba125, whose right inverted repeat (IRR) serves as a functional promoter. This promoter, particularly the -35 region, contributes to the regulation of *bla*_NDM_ expression [8, 9]. However, *bla*_NDM_ expression can originate from an intrinsic promoter upstream of the *bla*_NDM_ gene, functioning independently of insertion sequences such as ISAba125 [10]. Consistent with this observation, our previous analysis of the plasmids pEC135 and pEC150 demonstrated that the upstream region carrying the *bla*_NDM_ promoter does not include any contribution from insertion sequence elements. Instead, the promoter originates entirely from the *bla*_NDM_ gene itself [11]. These findings reveal the diversity of regulatory elements in *bla*_NDM_ upstream regions, ranging from intrinsic promoters to insertion sequences, highlighting the functional significance of non-coding sequences in *bla*_NDM_-mediated resistance. Building on these findings, our study aimed to identify conserved regulatory elements within the upstream regions of *bla*_NDM_ variants. Through the sequence analysis of 109 clinical isolates, we identified a conserved 110 bp sequence upstream of the *bla*_NDM_ gene. This sequence was consistently present across different bacterial strains, including *Escherichia coli*, *K. pneumoniae*, and *Enterobacter cloacae*. We further analyzed 2,706 *bla*_NDM_-positive sequences from the NCBI database, covering 80 bacterial species, to validate its conservation across NDM variants. Bioinformatics analysis confirmed the presence of the conserved region in all available 26 distinct NDM variants. Within the sequence, we also identified a core promoter (PNDM) and two transcription factor binding sites, ArcA and ArgR2. Site-directed mutagenesis of the PNDM promoter and the ArcA and ArgR2 binding sites demonstrated their critical roles in regulating *bla*_NDM_ expression and carbapenem resistance. The findings provide new insights into the regulatory mechanisms controlling *bla*_NDM_ expression and suggest potential therapeutic targets for combating carbapenem resistance.

## Materials and Methods

### Bacterial Strains

A total of 109 non-duplicated NDM-producing *Enterobacter*iaceae isolates were collected from clinical specimens at Zhejiang University Lishui Hospital between 2012 and 2023. Non-duplicated isolates are defined as unique bacterial strains obtained from distinct patients, clinical specimens, or time points, ensuring unbiased and independent data for analysis. These isolates were selected based on confirmed carbapenem resistance by antimicrobial susceptibility testing and molecular detection of the *bla*_NDM_ gene. The isolates encompassed diverse specimen types including blood, urine, sputum, and drainage fluid, representing various infection sites. The isolates included *E. coli* (*n* = 41), *K. pneumoniae* (n =28), *E. cloacae* (*n* = 14), *Citrobacter freundii* (n =7), *Enterobacter aerogenes* (n =3), and *Klebsiella aerogenes* (*n* = 3), among other species (*n* = 13). Species identification was initially performed using the VITEK2 Compact system (bioMérieux Vitek, USA), and results were confirmed by matrix-assisted laser desorption ionization time-of-flight (MALDI-TOF) mass spectrometry (bioMérieux Vitek, USA). The presence of the *bla*_NDM_ gene was verified by Sanger sequencing. For all isolates, genetic context analysis including plasmid typing and multilocus sequence typing (MLST) was performed to characterize the *bla*_NDM_ genetic environment.

### Data retrieval and Sequence Collection

As of October 8, 2024, *bla*_NDM_ gene sequences were retrieved from the NCBI Nucleotide database. The NDM CDS sequence was used as the query sequence against the nucleotide collection (nr/nt) database. The BLAST parameters were as follows: optimize for highly similar sequences (megablast) with an expect threshold of 0.05. This initial search yielded 4,162 sequences. Using Biopython, we extracted sequences containing the *bla*_NDM_ gene plus 110 bp of their upstream regions. The 110 bp upstream region of *bla*_NDM_ was selected for analysis because it encompasses the core promoter elements (-10 and -35 boxes) as well as transcription factor binding sites (ArcA and ArgR2). Sequences with incomplete *bla*_NDM_ CDS or upstream regions shorter than 110 bp were excluded, leaving 2,706 sequences for the final analysis NDM variants were identified by examining the "note" field within the CDS in GenBank annotations, and organism information was extracted from the sequence annotations. All *bla*_NDM_-positive sequences retrieved from the NCBI database were manually checked to ensure annotation accuracy and consistency.

### Shannon Entropy Analysis

To assess the conservation of the 110 bp upstream sequences, Shannon entropy (H) was calculated for each nucleotide position. Entropy quantifies the sequence variability, with lower values indicating higher conservation. For each position, i, entropy was calculated using the following formula:

H=−∑p(*i*)·log_2_(p(*i*))

Where p(*i*) represents the frequency of nucleotide *i* (A, T, C, G) at that position. Nucleotide frequencies were calculated across all sequences, with highly conserved positions showing entropy values near zero. This formula is derived from information theory and has been widely applied in genomic sequence analysis [12].

### Nucleotide Variation Analysis

Nucleotide variation analysis was performed to assess variability at each position within the upstream 110 bp region of *bla*_NDM_. The numbering of this region begins with position 1, corresponding to the nucleotide 110 bases upstream of the adenine (A) in the ATG start codon of *bla*_NDM_, with the adenine itself designated as position 111. Sequence alignments were conducted using Biopython, and the *bla*_NDM-1_ sequence (MH328008.1) served as the reference for this analysis. Clinical and database sequences were aligned to this reference to identify conserved and variable sites. At each nucleotide position, the most frequent base was defined as the reference nucleotide and variation rates were calculated by dividing the number of sequences containing an alternative base by the total number of sequences at that position. Positions with lower mutation frequencies were considered highly conserved, indicating dominance of the reference nucleotide, whereas higher mutation frequencies suggested greater variability. These nucleotide variation frequencies were used to quantify the conservation level within the upstream regulatory region.

### Evaluation of the *bla*_NDM_ Genetic Structures

Our previous study isolated and sequenced two plasmids, including pEC135 (Accession No. MH347484.1) from *E. coli* harboring *bla*_NDM-5_ and pEC150 (Accession No. MH328008.1) from *E. cloacae*, harboring *bla*_NDM-1_ [11]. The analysis also included p3R-IncX3 (Accession No. CP049352), which was retrieved from the NCBI database. The complete nucleotide sequences of pEC135 and pEC150 were determined using Sanger sequencing and these fully characterized plasmids, along with p3R-IncX3, served as templates for our current analyses of *bla*_NDM_ genetic structures.

### Promoter Prediction and Mutagenesis

The putative promoter region within the 110 bp sequence upstream of the *bla*_NDM_ gene was initially predicted using the online software, BPROM (Softberry), with a linear discriminant function (LDF) threshold of 0.2. The software was used to identify the -10 and -35 boxes of the potential promoter and to predict transcription factor binding sites within the conserved sequence. To delve into the regulatory role of that upstream region, the genetic structures of *bla*_NDM-1_ and *bla*_NDM-5_, along with their respective upstream regions, were amplified and cloned into the pET28X vector. A His-tag was added to the 3' end of the open reading frame (ORF) to create fusion protein expression plasmids, which were subsequently used as templates for mutagenesis experiments. The pET28X vector was constructed as previously described [13]. Briefly, pET28X is a modified version of pET28a (accession number LQ021053) where the T7 promoter region (positions 341 to 390) was deleted using SPC-PCR [14]. This modification allows for the insertion of gene structures between EcoRI and SalI restriction sites without interference from the vector's promoter, enabling the study of native promoter activities.

Specific mutations in the predicted promoter region were introduced using overlap PCR or single-primer cycle (SPC)-PCR [7]. The alterations were designed to disrupt key regulatory elements, including the promoter and transcription factor binding sites. Mutants of the PNDM promoter were generated to assess their impact on *bla*_NDM_ expression. Transcription factor binding site mutants targeting arcA and argR2 were constructed using the pET-28X-NDM5-His plasmid as the template. The *E. coli* Trans1-T1 (ECT1) and *K. pneumoniae* ATCC 13883 (KP13883) were used as the recipient strains for plasmid transformations.

### Electrophoretic Mobility Shift Assay (EMSA)

DNA fragments containing wild-type or mutant binding sites were amplified from three different plasmids: pET28X-NDM5-His (containing wild-type NDM-5 with intact transcription factor binding sites), pET28X-MarcA-NDM5-His (harboring mutations in the ArcA binding site), and pET28X-MargR2-NDM5-His (harboring mutations in the ArgR2 binding site). All fragments were amplified using primers NDM-US-F (ATGGCTTTT GAAACTGTCGC) and NDM-US-R (GCATCAATGCAGCGGCTAAT). Purified ArcA and ArgR2 proteins were purchased from CUSABIO (China). For binding reactions, 4 μg of recombinant protein was initially incubated with binding buffer (100 mM Tris-HCl pH 7.5, 10 mM MgCl2, 2 mM DTT, 100 mM KCl, 10% glycerol) on ice for 5 min. The DNA fragments were then added to the reaction mixture and incubated at 37°C for 30 min. The protein-DNA complexes were separated on a 1.5% TAE agarose gel at 120 V for 40 min. The gels were visualized using a gel imaging system (Hitachi High-Technologies, Japan).

### Susceptibility and MIC Testing

The MICs of imipenem (IPM), meropenem (MEM), piperacillin-tazobactam (TZP), cefepime (FEP) and aztreonam (ATM) were determined for *E. coli* and *K. pneumoniae* using the broth microdilution method. All MIC tests were performed in triplicate to ensure reproducibility. MIC interpretations were based on the guidelines established by the Clinical and Laboratory Standards Institute (CLSI). *E. coli* ATCC 25922 served as the quality control strain.

### Quantitative Real-Time PCR (qRT-PCR)

Total RNA was extracted from *E. coli* transformants using the TransZol Up Plus RNA Kit (TransGen Biotech, Beijing). RNA concentration and purity were determined using a NanoDrop 2000 spectrophotometer (Thermo Fisher Scientific, USA). Approximately 1 μg of total RNA was reverse transcribed to cDNA using FastKing gDNA Dispelling RT SuperMix (TIANGEN Biotech). Quantitative PCR (qPCR) was performed on an ABI 7500 Real-Time PCR System using the PerfectStart Green qPCR SuperMix (both from Applied Biosystems, USA). *bla*_NDM-5_ mRNA expression levels were normalized to 16S rRNA expression. The *bla*_NDM-5_-specific primers were qNDM-F (5'-GGCAGCACACTTCCTATCTC-3') and qNDM-R (5'-CGACAACGCATTGGCATAAG-3'). Primers for the 16S rRNA gene and the detailed reaction conditions were as previously described [13]. Statistical analyses were performed using GraphPad Prism 8 (GraphPad Software, USA) with Student's *t*-tests.

### Western Blot

Recombinant plasmids with a His-tag at the C-terminal region of the *bla*_NDM_ gene were transformed into *E. coli*. The transformants were cultured in Luria-Bertani (LB) medium, harvested, and lysed to extract total proteins. Protein concentrations were quantified using a fully automated biochemical analyzer (Hitachi 7600-020, Hitachi High-Technologies). *bla*_NDM_ production was evaluated by immunoblotting to detect the expression of the His-tagged protein. Experiments were conducted in triplicate, with results normalized to the housekeeping protein, DnaK, to ensure inter-sample consistency.

### Statistical Analysis

Statistical analyses were performed using GraphPad Prism 8 (GraphPad Software, USA). Data are presented as the mean ± standard deviation (SD). All results, with a *P* < 0.05 considered to indicate statistical significance.

## Results

### Characterisation of NDM-Positive Clinical Isolates

A total of 109 *bla*_NDM_-positive clinical isolates were analyzed, revealing two predominant variants: *bla*_NDM-1_ (65 strains, 59.6%) and *bla*_NDM-5_ (44 strains, 40.4%). Those isolates primarily belonged to *E. coli* (37.6%), *K. pneumoniae* (25.7%), and *E. cloacae* (12.8%). Notably, 82.6% of the strains harbored IncX3-type plasmids, which are likely to facilitate the transfer of the *bla*_NDM_ gene. Thus, the findings demonstrate that NDM-1 and NDM-5 are the predominant variants in current clinical practice, laying the foundation for future studies on conserved sequences upstream of the *bla*_NDM_ gene.

### Conservation of the 110 bp Upstream Sequence in *bla*_NDM_ Genes

**Fig. 1:**
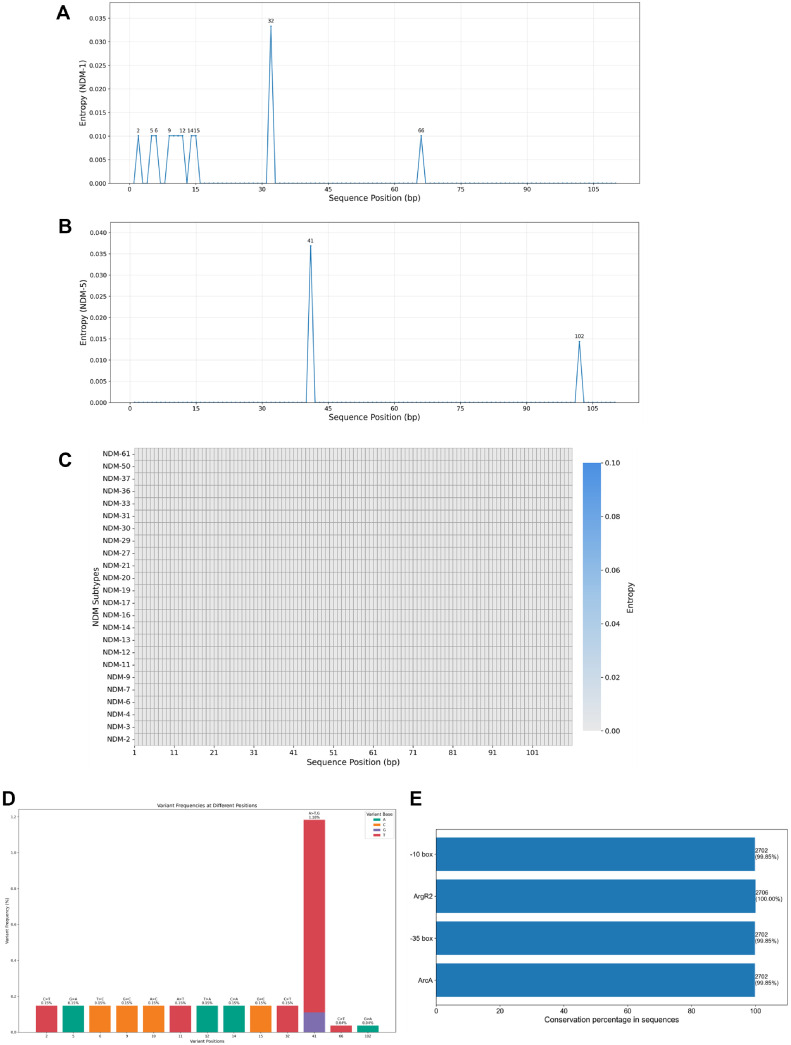
The 110 bp conserved sequence upstream of *bla*_NDM_ genes. (**A**) Entropy analysis of the 5' upstream sequence of the *bla*_NDM-1_ gene. (*n* = 1,154). Peaks indicate minor variations in the highly conserved sequence. (**B**) Entropy analysis of the 5' upstream sequence of the *bla*_NDM-5_ gene. (*n* = 768). (**C**) Heatmap of nucleotide entropy of the upstream sequences of other *bla*_NDM_ variants. Higher color intensity represents greater variation. (**D**) Nucleotide variations in the 110 bp upstream sequence of *bla*_NDM_ genes. Each column represents a specific mutation site, with column height indicating the variation frequency (*n* = 2,706). (X-axis not to scale). (**E**) Counts and percentages of features in the upstream sequence across all analyzed sequences.

Sequencing of 109 clinical isolates revealed a highly conserved 110 bp region upstream of the *bla*_NDM_ gene. Analysis of 2,706 *bla*_NDM_-positive sequences from the NCBI GenBank database showed that this conserved region was present across 26 distinct NDM variants. Entropy analysis of 1,154 *bla*_NDM-1_ and 768 *bla*_NDM-5_ sequences (Fig. 1A and 1B) indicated high conservation, with most positions showing entropy values of 0. Only two positions exhibited minor variability: position 32 in *bla*_NDM-1_ (entropy value 0.033) and position 41 in *bla*_NDM-5_ (entropy value 0.037). Eleven other positions across both variants exhibited minimal variability, with a median entropy value of 0.010. Entropy analysis of the remaining 24 NDM variants (Fig. 1C) revealed complete conservation across all positions, further confirming the stability of the 110 bp upstream region. These findings confirm the high degree of conservation in the 110 bp upstream sequence, with only minor variations observed at specific sites in *bla*_NDM-1_ and *bla*_NDM-5_. Nucleotide variation analysis across the entire dataset (Fig. 1D) revealed that 88% (97/ 110) of the positions were fully conserved. The most frequent variation occurred at position 41, with a mutation rate of 1.18%, while other variable positions had a median mutation rate of 0.15%. Notably, each variable position exhibited only a single type of nucleotide change. Examination of regulatory elements within the 110 bp upstream sequence (Fig. 1E) revealed that the ArgR2 binding site of the promoter was 100% conserved across all sequences, while the -10 box, -35 box, and ArcA binding site exhibited minimal variation, with only 0.15% of sequences containing mutations in these regions.

**Fig. 2:**
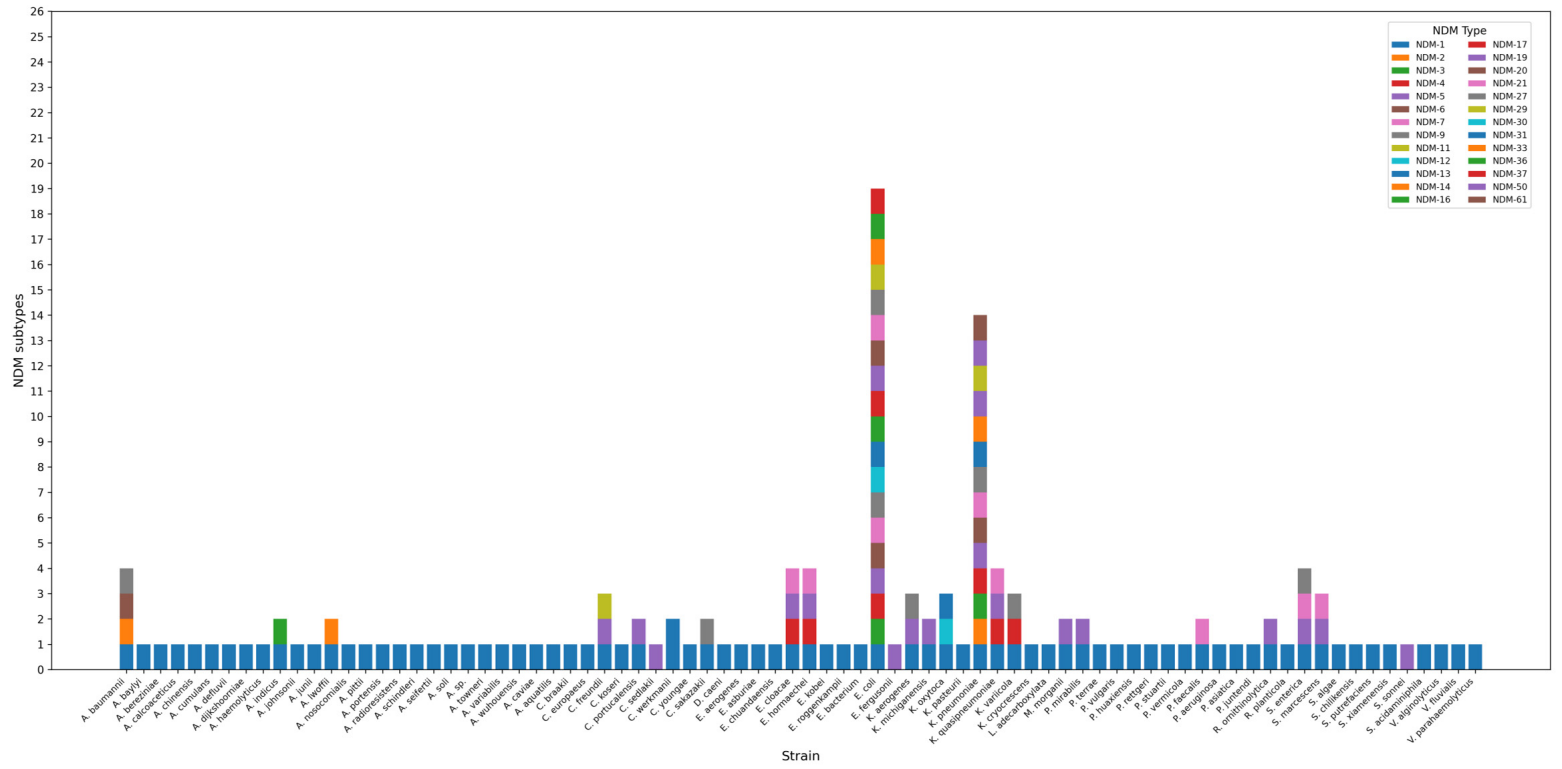
Distribution of *bla*_NDM_ variants and their upstream conserved 110 bp sequences across bacterial species. This figure shows the presence of conserved upstream sequences among 26 NDM variants in 80 species. Colored markers indicate the presence of different NDM variants in different strains.

Bioinformatics analysis confirmed that the conserved 110 bp upstream sequence was present in 2,706 sequences from 80 bacterial species, spanning all the available 26 NDM variants (Fig. 2). Among the top 10 bacterial species representing the majority of these sequences, the conservation rates of this region ranged from 99.89% to 100%. The regulatory elements, including the -35 box, -10 box, ArcA and ArgR2 binding sites, also showed high conservation, with minimal variations observed only in *K. pneumoniae* (Fig. S2). The results highlight the remarkable conservation of this region across diverse bacterial hosts and suggest its critical role in regulating *bla*_NDM_ expression.

### Plasmid Structure

**Fig. 3:**
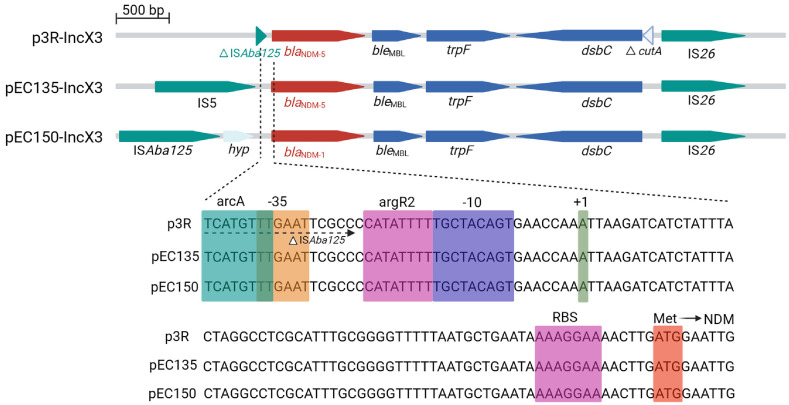
Comparative analysis of plasmid structures and the conserved upstream sequence of *bla*_NDM_. Schematic representation of three plasmids (p3R-IncX3, pEC135-IncX3, and pEC150-IncX3) containing *bla*_NDM_ genes. In p3R-IncX3, part of the ISAba125 sequence is located within the 110 bp conserved region. In contrast, in pEC135-IncX3 and pEC150-IncX3, the ArcA binding site and the -35 region of the PNDM promoter are intrinsic to the *bla*_NDM_ gene and contain no sequences from insertion elements. The alignment of the 110 bp upstream sequences shows the conserved PNDM promoter (-35 and -10 boxes), the transcription start site (+1), transcription factor binding sites (ArcA and ArgR2), a ribosome binding site (RBS), and the start codon (Met). Created in https://BioRender.com.

Structural analysis of the pEC135, pEC150, and p3R-IncX3 plasmids confirmed the presence of an identical 813 bp CDS encoding NDM-1 in pEC150 and NDM-5 in both pEC135 and p3R-IncX3. All three plasmids also contained the conserved 110 bp upstream sequence (Fig. 3).

### Promoter and Regulatory Elements

The 110 bp conserved upstream sequence contains the PNDM core promoter, which includes the -10 (TGCTACAGT) and -35 (TTGAAT) boxes essential for RNA polymerase binding and transcription initiation. LDF analysis yielded a score of 4.81, indicating strong promoter activity. The transcription start site (+1) position was identified as an adenine (A) nucleotide, according to Li *et al*. [15]. Two putative transcription factor binding sites were identified within the 110 bp sequence: arcA (TCATGTTT) near the -35 region and argR2 (CATATTTT) between the -10 and -35 boxes (Fig. 3). These transcription factors were identified through BPROM analysis and selected for further investigation. Both ArcA and ArgR2 are known bacterial gene regulators [16, 17].

### Recombinant Plasmids

Building upon the previously identified pEC135 and pEC150 plasmids, which are naturally occurring, we systematically generated six additional plasmids in this study (Table 1). The new constructs included pET28X-NDM1-His, pET28X-NDM5-His, pET28X-MpNDM1-His, pET28X-MpNDM5-His, pET28X-MarcA-NDM5-His, and pET28X-MargR2-NDM5-His. Each plasmid was transformed into *E. coli* Trans1-T1 recipient cells and the transformations were confirmed by antibiotic screening and DNA sequencing. Quantitative analysis revealed no significant differences in the copy number of the *bla*_NDM_ gene among the transformants harboring those plasmids (data not shown).

### MIC Testing

Susceptibility testing revealed significant differences in antibiotic resistance between the wild-type and mutant strains. Mutations in the PNDM promoter led to a marked reduction in the MICs for imipenem (IPM) and meropenem (MEM). The MICs of MpNDM1 and MpNDM5 strains decreased to 0.5 μg/ml and ≤0.25 μg/ml, respectively, compared to 256 μg/ml in the wild-type strains. A similar decrease in resistance was observed for cefepime (FEP) and piperacillin-tazobactam (TZP), with MICs dropping from ≥256 μg/ml to ≤1 μg/ml and ≥1024/4 μg/ml to 16/4 μg /ml, respectively (Table 2).

In contrast, among the transcription factor binding site mutations in *E. coli* Trans1-T1, ECT1-MargR2-NDM5 showed a reduction in carbapenem resistance, with MICs decreasing from 256 μg/ml to 16 μg/ml for IPM and to 32 μg/ml for MEM. The ECT1-MarcA-NDM5 strain showed less reduction than ECT1-MargR2-NDM5, with MICs decreasing to 64 μg/ml for both carbapenems. For both mutations, the MICs of cephalosporins and piperacillin-tazobactam remained at wild-type levels. Notably, all strains, both wild-type and mutant, consistently displayed low MIC values for aztreonam (ATM )(≤1 μg/ml )(Table 2).

To evaluate the conservation of ArcA and ArgR2 binding site effects across different bacterial species, we transformed the three plasmid constructs into KP13883. MIC results revealed that KP13883-MarcA-NDM5 and ECT1-MarcA-NDM5 maintained consistent susceptibility profiles. However, KP13883-MargR2-NDM5 exhibited higher MIC values for both IPM (64 μg/ml vs 16 μg/ml) and MEM (64 μg/ml vs 32 μg/ml) compared to ECT1-MargR2-NDM5 (Table 2).

### The mRNA Expression of the *bla*_NDM_ Gene

**Fig. 4:**
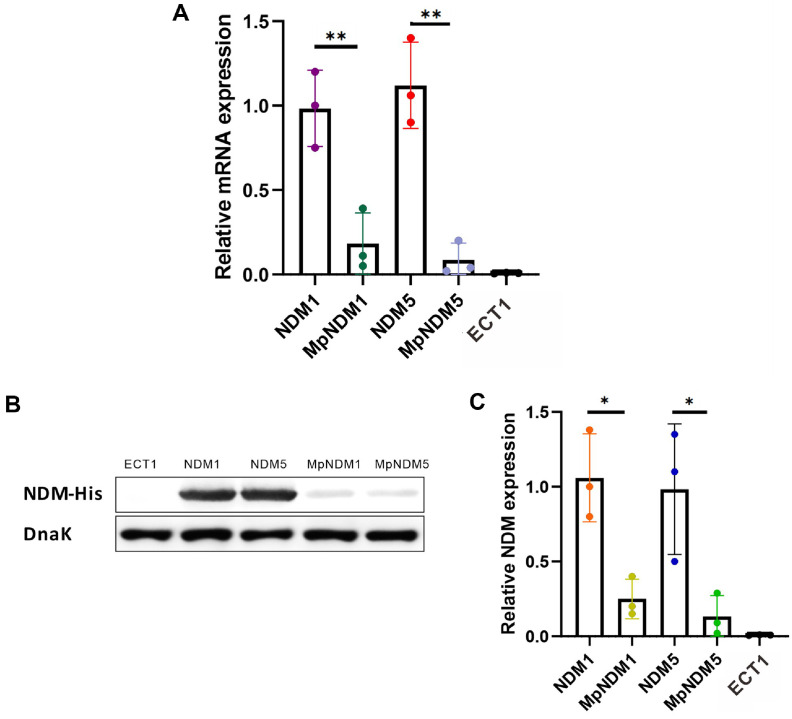
Effects of PNDM promoter mutations on carbapenem resistance, and mRNA and protein levels. (**A**) Relative *bla*_NDM_ mRNA levels measured by qRT-PCR. MpNDM1 and MpNDM5 showed significantly reduced mRNA levels, normalized to 16S rRNA. Data are presented as mean ± SD standardized to 16S rRNA. **P < 0.01, Student’s *t*-test. (**B**) Western blot analysis of NDM protein expression in wild-type and mutant strains. DnaK was used as the internal loading control. (**C**) Quantification of NDM protein levels, showing significant reductions in MpNDM1 and MpNDM5 strains. Data are presented as mean ± SD in NDM1 and NDM5 strains. *P < 0.05, Student’s *t*-test.

Quantitative PCR showed that mutations in the PNDM promoter significantly reduced *bla*_NDM_ mRNA expression in both NDM-1 and NDM-5 variants. Specifically, relative mRNA expression decreased by 90.27% in the MpNDM1 strain (*P* = 0.0087) and by 90.10% in the MpNDM5 strain (*P* = 0.0028) (Fig. 4A). Thus, the substantial reductions in mRNA levels underscore the critical role of the PNDM promoter in driving *bla*_NDM_ transcription.

Further analysis of the mutations in ArcA and ArgR2 transcription factor binding sites revealed differential effects on mRNA expression. The MarcA-NDM5 strain exhibited a modest reduction of 21.33%, which was not statistically significant (*P* = 0.34) (Fig. S1A). In contrast, the MargR2-NDM5 strain showed significant downregulation of *bla*_NDM-5_ mRNA levels by 75.33% (*P* < 0.01), suggesting that ArgR2 plays a more substantial role in transcriptional regulation than ArcA. However, both strains had a less pronounced impact on *bla*_NDM-5_ mRNA levels than PNDM promoter mutations.

### NDM Protein Expression

Western blot analysis confirmed that the reductions in mRNA expression due to PNDM promoter mutations translated into similar reductions in protein levels. In the MpNDM1 strain, NDM protein levels dropped by 94.57% (*P* = 0.0122), while in MpNDM5, protein expression decreased by 97.92% (*P* = 0.0326) (Fig. 4B and 4C). This consistency between mRNA and protein level reductions highlights the strong regulatory influence of the PNDM promoter on *bla*_NDM_ expression.

Mutations in the transcription factor binding sites also impacted NDM protein levels, however, to a lesser extent than promoter mutations. The MargR2-NDM5 strain exhibited a significant 32.67% decrease in protein expression (*P* = 0.0348), while the MarcA-NDM5 strain exhibited a lesser reduction of 24.00% (*P* = 0.0184)(Fig. S1C). These findings indicate that although ArcA and ArgR2 binding sites influence NDM protein production, mutations in the ArgR2 binding site had a more pronounced effect. However, neither transcription factor mutation affected protein expression as significantly as mutations in the PNDM promoter.

### Verification of the ArcA and ArgR2 Binding Sites

**Fig. 5:**
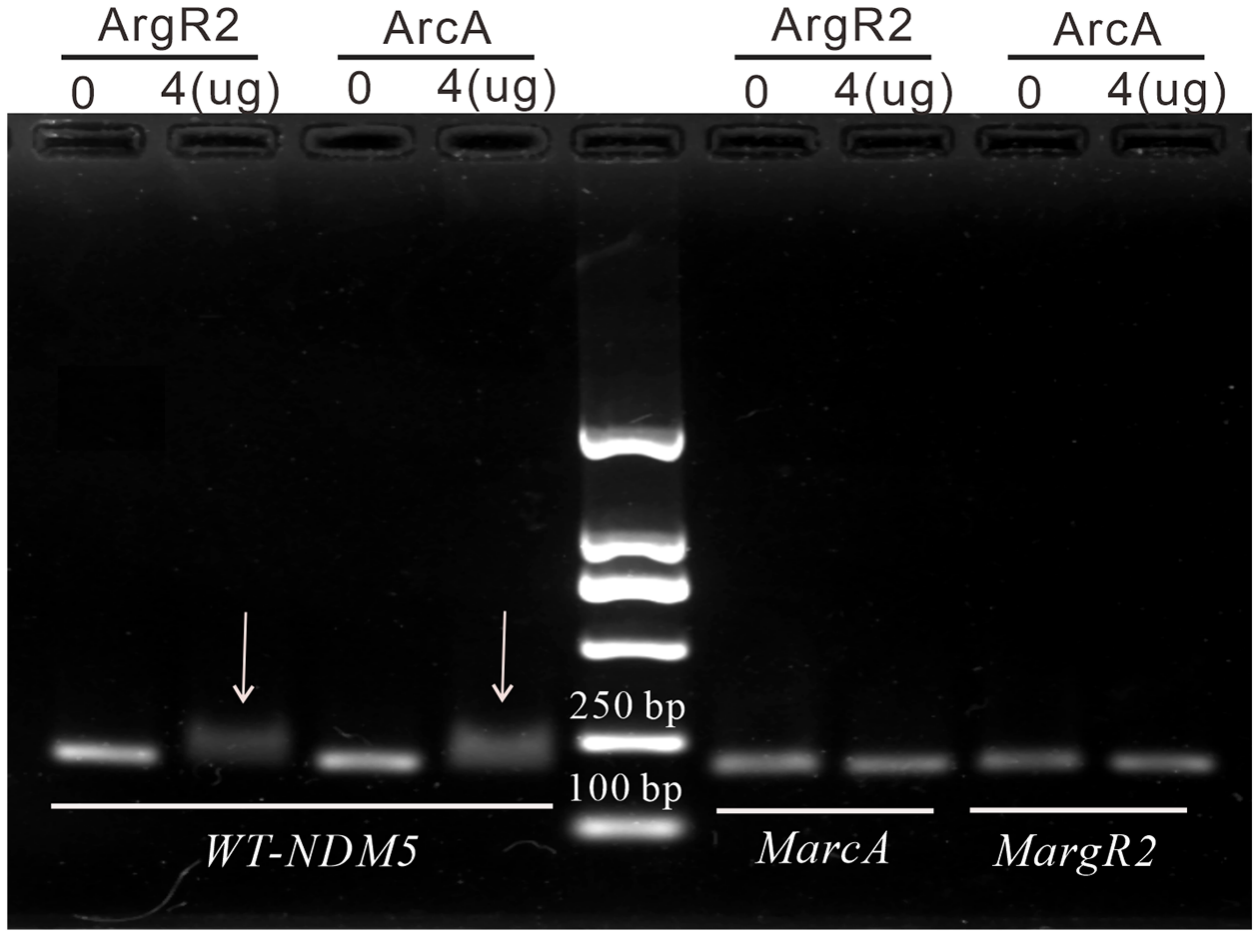
Verification of transcription factor binding to the predicted sites in the conserved 110 bp sequence. Electrophoretic mobility shift assay (EMSA) demonstrating specific binding of ArcA and ArgR2 transcription factors to their predicted binding sites. Left panel: wild-type DNA fragments (WT-NDM5) showed reduced mobility with 4 μg of ArcA or ArgR2 protein (indicated by white arrows), indicating protein-DNA binding. Right panels: Mutant DNA fragments (MarcA and MargR2) maintained the same mobility pattern with or without protein addition, confirming the specificity of the binding sites. The central lane contains DNA size markers with indicated sizes (250 bp and 100 bp).

EMSA results revealed that both ArcA and ArgR2 proteins (4 μg) bound specifically to the wild-type sequence, causing a visible mobility shift (Fig. 5). In contrast, DNA fragments with mutations in the respective binding sites (MarcA and MargR2) showed no mobility shift when incubated with the corresponding proteins. These findings confirmed that the predicted ArcA and ArgR2 binding sites within the conserved 110 bp sequence could be recognized and bound by their respective transcription factors *in vitro*.

## Discussion

This study identified a highly conserved 110 bp regulatory sequence upstream of the *bla*_NDM_ gene; this sequence was present across 80 bacterial species and 26 distinct NDM variants. Analysis of 2,706 *bla*_NDM_ sequences revealed that 88% of nucleotide positions within the 110 bp sequence were completely conserved, indicating strong selective pressure. This high conservation suggests the functional importance of the region and implies that it may contain essential regulatory elements, such as promoters [18]. Within the conserved sequence, we identified the PNDM core promoter and binding sites for two transcription factors, ArcA and ArgR2, both of which exhibited similarly high levels of conservation. The stability of these elements across a wide range of bacterial species and NDM variants highlights their potential as targets for novel strategies to combat NDM-mediated antibiotic resistance.

While research on amino acid substitutions and enzyme characteristics of new NDM variants has improved our understanding of NDM evolution, developing effective inhibitors against such enzymes remains challenging due to the growing diversity of variants [19]. Some inhibitors, including recently identified molecules such as pterostilbene and magnolol, exhibit reduced effectiveness against new NDM variants that contain key mutations [20-22]. In contrast to these challenges at the structural level, our study focused on regulatory sequences, identifying a conserved 110 bp upstream region that was present across 26 NDM variants and different bacterial species. Minor variations were found only in the NDM-1 and NDM-5 variants. Although upstream sequence data for other variants were unavailable, the conservation of the sequence across multiple bacterial hosts suggests it functions as a shared regulatory element essential for *bla*_NDM_ expression.

The exceptional conservation of key regulatory elements within the 110 bp sequence, particularly the -35 and -10 boxes, highlights their essential role in regulating *bla*_NDM_ expression. The PNDM promoter, with an LDF score of 4.81, exceeds that of the PY promoter (LDF: 4.06), which we identified in our previous study as the core promoter regulating *bla*_KPC_ expression in the Tn3-Tn4401 chimera [7]. Our experiments confirmed that mutations in the PNDM promoter significantly reduce resistance to β-lactam antibiotics by decreasing *bla*_NDM_ mRNA and protein levels. These findings align with previous studies showing that alterations in regulatory elements can reduce antibiotic resistance by disrupting essential gene expression [23].

Recent findings indicate that inhibiting the expression of resistance genes can restore antibiotic susceptibility. For example, transcriptome-guided antisense peptide nucleic acids (PNAs) have been shown to selectively inhibit gene expression, enhancing antibiotic efficacy [24, 25]. Similarly, peptide-conjugated phosphorodiamidate morpholino oligomers (PPMOs) have been used to suppress *bla*_NDM-1_ expression, restoring carbapenem susceptibility *in vitro* and *in vivo* [26, 27]. Thus, targeting the promoter region within the conserved 110 bp sequence presents a promising therapeutic approach. Specifically, constructing peptide-conjugated antisense oligonucleotides (ASOs) or PPMOs targeting this region may effectively suppress *bla*_NDM_ expression and restore bacterial susceptibility to carbapenems.

Although the transcription factor (TF) binding site mutations identified in our study affected *bla*_NDM_ expression, their impact was modest compared to promoter mutations. TFs typically recognize specific DNA sequences within promoters, modulating transcription by either enhancing or inhibiting its initiation and progression [28]. We identified two TF binding sites within the conserved sequence. ArcA is known to repress the multiple antibiotic resistance (*mar*) operon in Salmonella under anaerobic conditions, and ArgR2, a repressor involved in arginine metabolism [16]. Mutations in both transcription factor binding sites reduced IPM and MEM MIC values in ECT1 and KP13883 strains. However, ArgR2 binding site mutations in ECT1 demonstrated a more pronounced reduction in MIC values (16 μg/ml for IPM and 32 μg/ml for MEM) compared to ArcA binding site mutations (64 μg/ml for both antibiotics). In contrast, in KP13883, both mutations resulted in identical MIC values (64 μg/ml for IPM and MEM). These differences highlight that despite high conservation of transcription factor binding sites across bacterial species, their functional impact may vary in different genetic backgrounds. Before developing therapeutic strategies targeting these conserved regulatory elements, it is essential to evaluate their specificity and potential off-target effects. The species-specific variations in antimicrobial susceptibility observed in our study underscore the importance of functional validation across different bacterial hosts.

In summary, our findings provide new insights into the regulation of *bla*_NDM_ gene expression. We identified a highly conserved 110 bp regulatory sequence containing the PNDM core promoter and binding sites for ArcA and ArgR2 which plays a crucial role in *bla*_NDM_ expression. The sequence was largely conserved across diverse bacterial species and NDM variants, highlighting its potential as a target for novel inhibitors. Despite minor variations in the -35 box, the overall conservation of the PNDM promoter across various genetic contexts suggests its potential as an effective target for broadly inhibiting NDM expression, offering a promising approach to combatting NDM-mediated antibiotic resistance.

## Supplemental Materials

Supplementary data for this paper are available on-line only at http://jmb.or.kr.



## Figures and Tables

**Table 1:** Recombinant plasmids and their mutations.

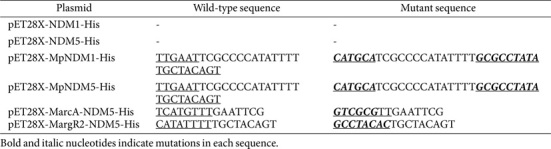

**Table 2:** MIC results for ECT1, KP13883 transformant strains, and their respective mutants treated with various antibiotics.