# Supplementary material for: Highly Conserved Genetic Factors Regulating blaNDM Gene Expression
Source: J Microbiol Biotechnol. 2025 Jul 14;35:e2412081. doi: 10.4014/jmb.2412.12081 (PMC12283253; doi:10.4014/jmb.2412.12081)
Supplement: Supplementary file 1 [file jmb-35-e2412081-supple.pdf]

J. Microbiol. Biotechnol. 2025. 35: e2412081
https://doi.org/10.4014/jmb.2412.12081

Supplementary Figures

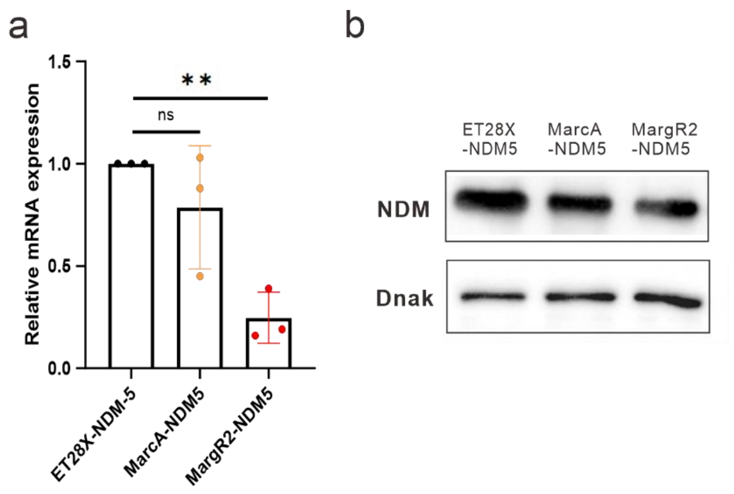

Fig. S1. Effects of ArcA and ArgR2 transcription factor binding site mutations on *bla*$_{\text{NDM-5}}$ mRNA and NDM protein expressions. (a) Relative *bla*$_{\text{NDM-5}}$ mRNA levels measured by qRT-PCR. MArgR2-NDM5 and MArcA-NDM5 show reduced mRNA expression compared to wild-type strains. Data are presented as the mean ± SD from three replicates. ns (non-significant); $^{**}P < 0.01$ (Student's t-test). **(b)** Western blot analysis of NDM protein expression in wild-type and mutant strains. DnaK was used as the internal loading control. **(c)** Quantification of NDM protein levels, showing reduced expression in MArgR2-NDM5 and MArcA-NDM5 strains. Data are the mean ± SD from three independent experiments. $^{*}P < 0.05$, $^{\#}P < 0.05$ (Student's *t*-test).

Bacterial Species	n	Overall Conservation (%)	-35 box (TTGAAT) Conservation (%)	-10 box (TGCTACAGT) Conservation (%)	ArcA binding site Conservation (%)	ArgR2 binding site Conservation (%)
Escherichia coli	1742	99.99	100.00	99.98	100.00	100.00
Klebsiella pneumoniae	360	99.95	99.63	100.00	99.79	100.00
Acinetobacter baumannii	130	99.89	100.00	100.00	100.00	100.00
Enterobacter hormaechei	55	100.00	100.00	100.00	100.00	100.00
Enterobacter cloacae	40	100.00	100.00	100.00	100.00	100.00
Pseudomonas aeruginosa	35	99.97	100.00	100.00	100.00	100.00
Citrobacter freundii	28	100.00	100.00	100.00	100.00	100.00
Proteus mirabilis	27	100.00	100.00	100.00	100.00	100.00
Providencia rettgeri	19	100.00	100.00	100.00	100.00	100.00
Salmonella enterica	19	100.00	100.00	100.00	100.00	100.00

Fig. S2. Conservation of the 110 bp upstream sequence of the *bla*$_{\text{NDM}}$ gene across different bacterial species. The 110 bp upstream region was analyzed across 2,706 sequences from 80 bacterial species. Only the top 10 species with the highest number of sequences are shown. Conservation percentage represents the proportion of invariant nucleotide positions within each region. The regions analyzed include the 35 box, -10 box, and the transcription factor binding sites for ArcA and ArgR2. High conservation values (100%) are highlighted in green.
